# Hospital-Acquired Infections Caused by *Acinetobacter baumannii*: A Comparative Analysis of Risk Factors with Other ESKAPE-E Pathogens in a Third-Level IMSS Hospital in Yucatan Mexico

**DOI:** 10.3390/diseases13120384

**Published:** 2025-11-26

**Authors:** Jael del Rosario Eb-Rejón, José Ramón Paniagua-Sierra, Carlos Gracida-Osorno, Gloria María Molina-Salinas

**Affiliations:** 1 División de Epidemiología, Hospital de Especialidades, CMN Ignacio García Téllez, Instituto Mexicano del Seguro Social, Mérida 97150, Yucatán, Mexico; jael.eb@imss.gob.mx; 2 Unidad de Investigación Médica en Enfermedades Nefrológicas, Hospital de Especialidades, CMN Siglo XXI, Instituto Mexicano del Seguro Social, Mexico City 06720, Mexico; ramon.paniagua@imss.gob.mx; 3 Servicio de Medicina Interna, Hospital General Regional No. 1, CMN Ignacio García Téllez, Instituto Mexicano del Seguro Social, Mérida 97150, Yucatán, Mexico; carlos.gracida@imss.gob.mx; 4 Unidad de Investigación Médica Yucatán, Hospital de Especialidades, CMN Ignacio García Téllez, Instituto Mexicano del Seguro Social, Mérida 97150, Yucatán, Mexico

**Keywords:** *Acinetobacter baumannii*, ESKAPE-E, healthcare-associated infections, risk factors, carbapenems

## Abstract

Background: *Acinetobacter baumannii* is classified within the ESKAPE-E group of pathogens, recognized for its role in causing severe infections, and is often associated with various healthcare-related infection (HAIs) types, particularly in intensive care units. This opportunistic pathogen is distinguished by its considerable antibiotic resistance and is associated with prolonged hospital stays, high medical costs, and increased mortality rates. Objective: This study investigated factors associated with HAIs caused by *A. baumannii*, versus other ESKAPE-E pathogens, to identify distinguishing intrinsic and extrinsic factors that guide the control and prevention of HAIs within our hospital. Methods: This study included patients from a Third-Level IMSS Hospital in Mérida, Mexico, between 2018 and 2022, with 54 cases (HAIs caused by *A. baumannii*) and 108 matched controls (HAIs caused by other ESKAPE-E pathogens). Results: Ventilator-associated pneumonia was the most frequent HAI in both groups, followed by catheter-related bloodstream infections. Comorbidities were more common in patients with HAIs caused by *A. baumannii* than in those with other ESKAPE-E pathogens. Most patients received antimicrobial treatment before HAIs development. Bivariate analysis showed that comorbidities and prior meropenem and linezolid treatment were significant risk factors, whereas multivariate analysis identified comorbidities and prior meropenem use as risk factors for *A. baumannii* HAIs versus other ESKAPE-E pathogens. Most *A. baumannii* isolates were extensively drug-resistant (90.7%), with 84% showing carbapenem resistance. Conclusions: This study highlights the importance of optimizing antimicrobial use and measures to mitigate *A. baumannii* HAIs. These findings have significant implications for infection control and antimicrobial stewardship in healthcare settings.

## 1. Introduction

Healthcare-associated infections (HAIs) are infections or toxin-related conditions that are absent upon admission but may emerge 48 h or more after hospital admission, or within 30 days of treatment. HAIs are the most prevalent adverse outcomes in the provision of healthcare services [1]. In 2023, a comprehensive systematic review of 400 studies conducted globally estimated the overall prevalence of HAIs to be 14% [2]. The World Health Organization (WHO) report on the global impact of HAIs indicates that infection rates range from 3.6% to 12% in high-income countries and from 5.4% to 19.1% in low- and middle-income countries [3]. A recent analysis by the Organization for Economic Co-operation and Development (OECD) and WHO projects that up to 3.5 million individuals worldwide could succumb to HAIs annually by 2050 [4].

Causal agents of HAIs include fungi, viruses, parasites, and bacteria [5], notably the ESKAPE-E bacteria, *Enterococcus faecium*, *Staphylococcus aureus*, *Klebsiella pneumoniae*, *Acinetobacter baumannii*, *Pseudomonas aeruginosa*, *Enterobacter* spp., and *Escherichia coli*, which are a major cause of HAIs globally and exhibit high rates of antimicrobial resistance (AMR) [6,7]. The WHO has categorized drug-resistant ESKAPE-E pathogens as critical, high-, and medium-priority pathogens, showing their substantial threat to human health, and the need for urgent research, development, and public health interventions [8,9]. Among this group, the critical priority pathogen *A. baumannii* is an opportunistic bacterium that infects critically ill patients and has emerged as a global health concern owing to its ability to survive in harsh environments such as medical equipment, contributing to the spread of multidrug-resistant (MDR) clones in hospitals. The management of *A. baumannii* HAIs is significantly complicated by this issue, leading to prolonged hospitalizations, and a high prevalence of drug resistance, often to carbapenems, which are regarded as last-resort antibiotics. Drug resistance of *A. baumannii* is associated with higher mortality rates [10,11].

In Mexico, HAIs caused by *A. baumannii* have emerged as a significant concern because of the high drug resistance rate of this bacterium to multiple antibiotics and its association with hospital outbreaks [12,13,14]. Single-center and multicenter investigations have reported drug resistance rates exceeding 50% for conventional antibiotics and 69–90% for carbapenems against *A. baumannii*, which are higher than the global averages [15,16,17,18]. In Yucatan, Mexico, our research group documented an increase in antibiotic resistance rates for *A. baumannii* in patients from the intensive care unit (ICU), highlighting carbapenem resistance, which increased from 56.6% (2016–2018) to 95.5% (2019–2021) [19,20].

Although investigations have explored the risk factors for *A. baumannii* infections across diverse patient populations [21,22,23], there is a paucity of studies focusing on the potential differences in risk factors between HAIs caused by *A. baumannii* and those caused by other ESKAPE-E priority pathogens. This study hypothesized that both intrinsic and extrinsic epidemiological and clinical factors contribute to the risk of acquired HAIs caused by *A. baumannii* in contrast to other ESKAPE-E bacteria. Consequently, this study aimed to investigate the factors associated with HAIs caused by *A. baumannii*, as opposed to the HAIs caused by other ESKAPE-E pathogens, in patients attending a Tertiary-Care Hospital in Merida, Yucatán, Mexico, and to identify distinguishing intrinsic and extrinsic factors that guide the control and prevention of HAIs within our hospital.

## 2. Materials and Methods

### 2.1. Study Type and Design

This study employed a retrospective case–control design conducted at the Instituto Mexicano del Seguro Social (IMSS) Yucatán Tertiary-Care Hospital, which includes 175 beds, spanning the period from 1 January 2018 to 31 December 2022. HAIs caused by ESKAPE-E pathogens were identified using clinical and microbiological criteria. The case group consisted of patients who developed some type of HAIs caused by *A. baumannii*, while the control group included patients who acquired some type of HAIs of bacterial etiology from the ESKAPE-E group, excluding *A. baumannii*, during their hospitalization. The selection criteria are listed in Table 1. The matching criteria for cases and controls were set at a 1:2 ratio, ensuring that each case and its corresponding control were matched by the same type of HAIs, sex, and similar age group.

### 2.2. Sample and Sampling Methodology

The sample size was determined using Epi Info 7.2 for Windows statistical software, specifically using the StatCalc function. The calculation resulted in 54 cases and 108 controls to maintain a 1:2 case–control ratio. A non-probabilistic sequential sampling technique was used.

### 2.3. Data Collection

Information was collected through a review of the Nosocomial Infections Registration System (INOSO, IMSS) and patient medical records without direct participant contact or intervention. Information about demographic, clinical, and microbiological data was extracted from medical and laboratory records.

### 2.4. Statistical Analysis

Data processing and tabulation were performed using Microsoft Excel, and statistical analyses were performed using SPSS version 25.0 (IBM, New York, USA). A comprehensive descriptive analysis was conducted for all the study variables. Qualitative variables were expressed as frequencies and percentages, and quantitative variables were expressed as means and standard deviations. Data were assessed using the Kolmogorov–Smirnov test. Student’s *t*-test was used for continuous variables, and the Mann–Whitney U test was used for non-normally distributed variables. Categorical variables were analyzed using the chi-squared and Fisher’s exact tests. The association between variables was measured using odds ratios (OR) with 95% confidence intervals (CI) of the variables, with statistical significance established at significance set at *p* < 0.05.

## 3. Results

### 3.1. HAIs Caused by ESKAPE-E

**Figure 1:**
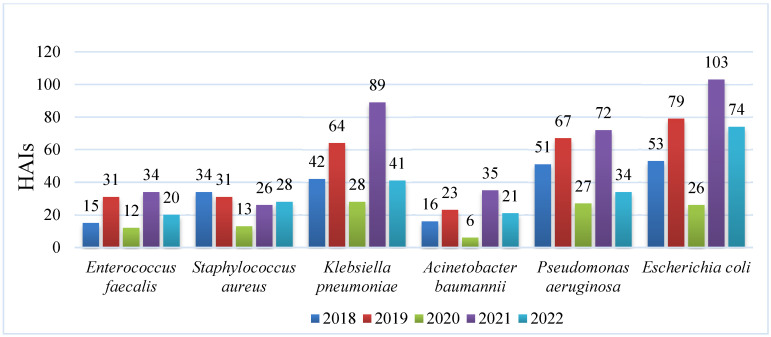
Frequency of healthcare-associated infections attributed to ESKAPE-E pathogens among patients at the IMSS Yucatán Third-Level Hospital between 2018 and 2022.

During the five-year study period, 1892 HAIs were documented. Among them, 101 cases of *A. baumannii* HAIs were identified, resulting in an overall prevalence of 5.3%. In contrast, 1094 HAIs were attributed to the rest of the ESKAPE-E pathogens, with a prevalence of 57.8%, and infection caused by *E. coli* was the most common (30.8%). No HAIs caused by *Enterobacter* spp. were identified during the study period (Figure 1).

**Figure 2:**
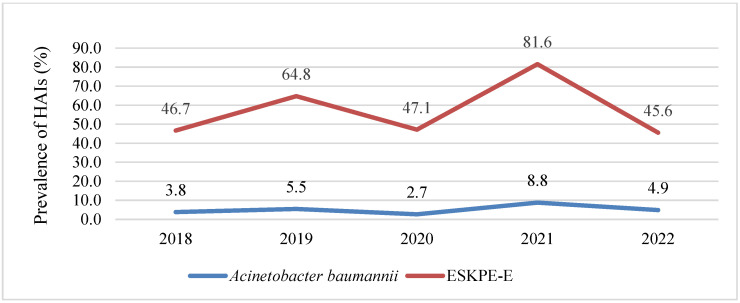
Prevalence of healthcare-associated infections caused by *A. baumannii* in comparison with those caused by the rest of the ESKAPE-E pathogens among patients at the IMSS Yucatán Third-Level Hospital from 2018 to 2022.

The annual prevalence of *A. baumannii* HAIs fluctuated from 2018 to 2022. The prevalence peaked in 2021 at 8.8% before decreasing to 4.9% in 2022, marking the highest value for the study period. A notable decrease was observed in 2020, which may be attributable to the initial wave of the COVID-19 pandemic. The most concerning aspect was the tripling of *A. baumannii* HAIs prevalence during the study period (2021 vs. 2022). A similar trend was observed for other ESKAPE-E bacterial HAIs, with higher prevalence rates in 2021 and similar rates in 2018, 2020, and 2022 (Figure 2).

### 3.2. Sociodemographic and Clinical Characteristics of Case and Control Groups and Univariate Analysis

Following the description of the distribution and prevalence of HAIs caused by *A. baumannii* in comparison to other ESKAPE-E pathogens, the sociodemographic and clinical characteristics of patients with HAIs caused by *A. baumannii* (cases) and those with HAIs due to other ESKAPE-E pathogens (controls) were examined. This analysis aimed to identify the risk factors associated with *A. baumannii* infection.

Table 2 delineates the sociodemographic and clinical characteristics of the case and control groups concerning HAIs. Considering that this was a matched case–control study based on age and HAIs type, it is noteworthy that the control group consisted of slightly younger participants. Both groups showed a notable prevalence of hypertension, diabetes mellitus, and oncohaematological diseases as comorbidities. The invasive procedure was more common, without statistical differences in patients with *A. baumannii* HAIs prior to mechanical ventilation (75.9% vs. 58.3%, *p* = 0.37) and exhibited prolonged hospital stays (40.2 vs. 36.9 days, *p* = 0.122) compared to the control group, whereas the average number of days in the ICU was almost the same for both groups (21.4 vs. 22.4 days, *p* = 0.196). Most patients in both groups were discharged from the hospital (57.4% vs. 60.2%, *p* = 0.360). HAIs caused by *A. baumannii* were associated with a mortality rate of 37.0%, which was slightly higher than the 28.7% mortality rate associated with HAIs caused by other ESKAPE-E microbes.

In accordance with Magiorakos et al. (2012) [24], we found that a substantial proportion of *A. baumannii* clinical isolates were classified as extensively drug-resistant (XDR; 90.7%), with 84% of them demonstrating carbapenem resistance. The most prevalent bacteria in the control group were *E. coli* (28.8%), *P. aeruginosa* (25.9%), and *K. pneumoniae* (24.0%). The MDR profiles ranged from 3.7% to 15.8%, and XDR profiles ranged from 2.8% to 24.1%, with higher prevalence observed in *E. coli* (24.1%) and *K. pneumoniae* (20.3%) [24]. In addition, the prior administration of fluoroquinolones, carbapenems, and third-generation cephalosporins was frequently observed before the onset of HAIs caused by *A. baumannii.* Conversely, the use of third-generation cephalosporins, fluoroquinolones, and aminoglycosides was more common in HAIs caused by other ESKAPE-E pathogens. In the case group, the prescription of three antibiotics was the most common (31.5%), while in the control group, one antibiotic was prescribed (47.3%).

Furthermore, univariate analysis identified statistically significant differences between the groups in terms of comorbidities, duration of invasive procedures, and prior antibiotic therapy with fluoroquinolones, carbapenems, and oxazolidinones.

### 3.3. Risk Factors Associated with HAIs Caused by A. baumannii vs. Other ESKAPE-E Pathogens

Table 2 summarizes the patients characteristics and risk factors associated with HAIs caused by *A. baumannii* compared to those caused by other ESKAPE-E pathogens. Bivariate analysis suggested an association with comorbidities and prior treatment with levofloxacin (LEV), meropenem (MEM), and linezolid (LZD). Given the potential influence of confounders, a conditional logistic regression was performed to adjunt for covariates such as age, sex, comorbidities, and invasive procedures. This adjustment allowed for the identification of independent predictors. The multivariable model confirmed that comorbidities and MEM use were significantly associated with *A. baumannii* HAIs, indicating that these variables exert an independent effect beyond overlapping clinical conditions or treatment exposures.

## 4. Discussion

The findings confirmed that intrinsic factors, such as comorbidities, and extrinsic factors, such as the duration of invasive devices and prior antimicrobial treatment, increase the risk of *A. baumannii* HAIs.

### 4.1. Sociodemographic and Clinical Characteristics

The median age of the patients was 41 years and that of the controls was 46 years, which differs from studies reporting median ages of 59 and 60 years. This discrepancy likely stems from the inclusion of patients from various departments, not only the ICU [25,26]. The mean age was 39.26 years (SD ± 26.6) for cases and 39.8 years (SD ± 26.8) for controls, contrasting with studies reporting means of 55.13 years (SD ± 18.44) for cases and 58.47 years (SD ± 19.77) for controls [27], as those focused on adults and specific HAIs. Furthermore, most patients in each group were predominantly within the age ranges of 27–59 years (38.9% vs. 37%, *p* = NS) and ≥60 years (31.5% vs. 29.6%, *p* = NS). Both groups showed higher male predominance than female predominance (83.3 vs. 16.7%, *p* = NS), consistent with Huertas Vaquero et al. (2021) [26], who reported high prevalence of male cases (65% cases involving HAIs caused by *A. baumannii*) compared to controls (53.4% without infection), in contrast to female cases (35% cases) compared to controls (46.6%), which were derived from matching based on HAIs type and sex, in contrast to previous studies [28]. Factors influencing sex-based infection outcomes include the quality of medical care, sex hormones, immune responses from sex-related polymorphisms, and chronic comorbidities [28]. Further research is needed to investigate these sex differences in HAIs caused by ESKAPE-E including the enhancement of pro-inflammatory cytokines by estradiol [29]. Monitoring blood glucose levels is essential because hyperglycemia increases the risk of mortality, complications, and HAIs [30]. Infections caused by *A. baumannii* are more prevalent in patients with preexisting health conditions or chronic illnesses [31,32]. In our study, 88.9% of the case group presented with at least one health issue upon admission, in contrast to 72.2% of the control group. Ardoino et al. (2016) [21] found trauma, ulcers, malnutrition, and liver diseases to be common health issues in patients with HAIs caused by *A. baumannii*. However, our findings showed that hypertension, diabetes, hematological malignancies, and low body weight were the most prevalent conditions. The first three conditions were observed in both groups. This discrepancy is likely attributable to our hospital’s specialization in oncology and the higher prevalence of chronic diseases in our country compared to Europe. Malnutrition affects 25–50% of hospitalized patients and often worsens during hospitalization. Given the link between nutrition, infection, and immunity, future research should examine the nutritional status of patients with hospital-acquired infections [33,34].

Among the 54 patients (cases), 36 acquired HAIs due to *A. baumannii* during their ICU stay. Conversely, 68 of the 108 controls developed HAIs attributable to other ESKAPE-E bacteria in the ICU (62.9 vs. 66.7%). These findings underscore the high infection rates in the ICU and the challenges in managing critically ill patients who require invasive devices. For *A. baumannii* HAIs, our results for ventilator-associated pneumonia (VAP) at 50% and catheter-related bloodstream infections (CRBSI) at 29.6% are consistent with national data, which report VAP and central line-associated bloodstream infection rates of 57.9% and 16.8%, respectively [35]. In the United States, HAIs caused by *A. baumannii* account for 6% of VAP cases and 2% of CRBSI cases [36]. The analysis of invasive device use in our study indicated that the case group had the highest incidence of interventional medical procedures, with the central venous catheter (CVC) being the most frequently used device, employed in 98.1% of cases and 89.8% of controls. This finding contrasts with the results reported by Gu et al. (2021) [27], who identified tracheal intubation as the most common procedure among cases (48.9%) and controls (50.0%), and by Ardonio et al. (2016) [21], who documented endoscopy as the predominant invasive procedure in their case group (77.9%) and controls (25.0%). CVCs are often used in patients from highly specialized hospitals, such as those in our study, who are undergoing treatment for severe conditions and require a patent central line for antibiotic administration and fluid therapy.

According to Playford et al. (2007) [25], 52% of patients had HAIs caused by carbapenem-resistant *A. baumannii*. The primary infections were VAP, bloodstream infection, and surgical site infection, with 19, 7, and 4 reported cases, respectively. The drug resistance profiles of *A. baumannii* in our study were consistent with those reported in international and national studies. The frequency of MDR (35.4%) and extensively drug-resistant (XDR, 7.1%) profiles among isolates in Central America [37]. In South Africa [38], the ranges of MDR, XDR and pan-resistant (PDR) were 53–60% for MDR, 1–19% for XDR, and 1% for PDR, respectively. The National Institute of Cancerology of Mexico City reported 24% MDR *A. baumannii* in blood cultures [39], whereas Christus Muguerza Hospital in Monterrey, Mexico reported 64% XDR *A. baumannii* in ICU patients [40]. In our study, XDR *A. baumannii* was identified as the most prevalent resistance profile, accounting for 90.7% of cases, while the remaining 9.3% were classified as MDR. AMR has emerged as a significant phenomenon that increases global healthcare costs. Despite the development of new antimicrobial agents, AMR continues to increase [41]. The GEIH-REIPI-Ab 2010 project in Spain reported *A. baumannii* drug resistance rates exceeding 94% for ceftazidime, piperacillin, and ciprofloxacin; 82–86% for carbapenems and tetracycline; and 60–70% for tobramycin, sulbactam, gentamicin, and doxycycline. The drug resistance rates were 49%, 30%, 24%, and 3% for amikacin, minocycline, tigecycline, and colistin, respectively [42]. Previous AMR reports from our hospital’s ICU showed resistance to third- and fourth-generation cephalosporins (81.13–67.92%), ciprofloxacin (79.25%), gentamicin (84.91%), and trimethoprim/sulfamethoxazole (77.36%) [19]. Our research showed that AMR increased over five years, with resistance to ceftazidime and ciprofloxacin rising from 83% to 100%, cefepime and trimethoprim/sulfamethoxazole from 79% to 100%, gentamicin from 64% to 100%, and carbapenems from 33% to 74%. The efficacy of carbapenems has been increasingly undermined by resistance mechanisms [36,43]. This increase in AMR limits therapeutic options and facilitates the spread of drug-resistant pathogens, which is attributed to the selective pressure from inappropriate antimicrobial use [44,45].

Regarding the administration of antibiotics prior to any type of HAIs, Huertas-Vaquero et al. (2021) [26] reported that all patients had received prior antibiotic therapy, in contrast to only one in three control patients. The antibiotics used in both groups included ceftazidime, ceftriaxone, amoxicillin/clavulanate, imipenem, LEV, LZD, and vancomycin [17]. Gu et al. (2021) [27] observed a significantly higher frequency of prior carbapenem use in their case group (83.0%) compared to the controls (26.6%). In our study population, 100% of patients in the case group received antibiotic therapy prior to HAIs, with the most frequently prescribed antibiotics being MEM (15.3%), LEV (12.5%), and vancomycin (9.7%). In contrast, 91.6% of the control patients had received prior antibiotic therapy, predominantly cefotaxime (14.4%), LEV (12.3%), and amikacin (10.7%).

Evaluating the clinical impact of *A. baumannii* on mortality is challenging because of its tendency to infect critically ill patients who often experience adverse outcomes regardless of infection. Mortality rates associated with *A. baumannii* range from 26% to 68%, although directly attributing deaths continue problematic [25]. We observed higher mortality in cases than in controls (37.0% vs. 28.7%). Gu et al. (2021) [27] reported 34.0% mortality in patients with HAIs from *A. baumannii*, compared to 21.0% for other site infections. Ren et al. (2019) [46] identified 19.4% mortality in patients with hospital-acquired pneumonia due to MDR *A. baumannii* versus 6.1% in those with susceptible *A. baumannii*.

### 4.2. Risk Factors

Analysis of the groups revealed statistically significant differences in comorbidities, duration of invasive procedures, and prior antibiotic treatments with fluoroquinolones, carbapenems, and oxazolidinones (Table 2). The initial bivariate analysis revealed significant associations for some variables, such as comorbidities (OR 3.07, 95% CI 1.19–7.93, *p* = 0.016), and prior use of one-by-one antibiotics was identified as a risk factor for MEM (OR 7.56, 95% CI 3.16–18.08, *p* < 0.0001) and LZD (OR 6.53, 95% CI 2.19–19.49, *p* < 0.0001). Subsequent multivariate analysis revealed that: (1) comorbidities increased the probability three-fold of acquiring any type of HAI by *A. baumannii* compared to other ESKAPE-E microbes, and (2) prior use of MEM increased the likelihood of acquiring HAIs by *A. baumannii* 5.47-fold (2.12–14.07 CI, *p* < 0.0001). These findings align with those of prior studies, such as that by Huertas Vaquero et al. (2021) [26], which identified previous carbapenem use as having an OR of 4.26 (95% CI, 2.52–5.90, *p*= 0.001) and quinolones with an OR of 3.15 (95% CI 1.20–5.10, *p* = 0.002) as risk factors for *A. baumannii* infection and/or colonization. Ardonio et al. (2016) [21] also supported this by demonstrating that carbapenem use (OR 11, 95% CI 1.42–85.19, *p* = 0.021) was linked to the onset of *A. baumannii* infection. It is essential to examine antibiotic usage, especially regarding duration and dosage, in the context of *A. baumannii*, which arises from the pressure of broad-spectrum antibiotics and transmission of strains among patients, healthcare staff, and hospital settings [47,48]. In our Tertiary-Care Hospital, patients often need extended stays and prolonged use of invasive devices. If these devices are not correctly installed and maintained, there is an increased risk of infection by complex bacteria such as *A. baumannii*.

## 5. Conclusions

Our findings indicate that the presence of comorbidities, the number of days of utilization of invasive devices, and prior prescription of carbapenems predispose patients (cases) to developing HAIs caused by *A. baumannii*, in contrast to patients (controls) with the same type of HAIs caused by other members of the ESKAPE-E pathogens. Early identification of risk factors, such as comorbidities that predispose individuals to infection by *A. baumannii* rather than other ESKAPE-E bacteria, is essential. The appropriate use and maintenance of medical devices, along with the judicious use of antibiotics, particularly MEM, are critical for mitigating and managing the escalation of AMR. Enhanced regulatory measures governing antibiotic prescriptions are essential to reduce unnecessary usage. This is particularly important for carbapenems, which should be reserved for use as a last resort. Therefore, it is imperative to strengthen antibiotic stewardship programs.

## Figures and Tables

**Table 1:** Selection criteria for cases and control groups.

Selection Criteria	Cases	Controls
Inclusion	- Patients with a hospital stay exceeding 48 h - Patients with HAIs caused by *A. baumannii* during the study period - Availability of antibiogram	- Patients with a hospital stay exceeding 48 h - Patients with HAIs caused by the other ESKAPE-E bacteria during the study period - Availability of antibiogram
Exclusion	- Patients with healthcare-associated infections developed outside the hospital - Patients with healthcare-associated infections by multiple microorganisms - Patients experiencing reinfection or recurrence	- Patients with healthcare-associated infections developed outside the hospital - Patients with healthcare-associated infections by multiple microorganisms - Patients experiencing reinfection or recurrence

**Table 2:** Sociodemographic and clinical characteristics of patients and risk factors associated with healthcare-associated infections caused by *A. baumannii* vs. those caused by the other ESKAPE-E pathogens.

Sociodemographic and Clinical Characteristics	*A. baumannii* *n* (%)	ESKPE-E *n* (%)	*p*-Value	OR
Male	45 (83.3%)	90 (83.3%)	1.000	
Avg. Age (years)	39.2 SD ± 26.6	39.8 SD ± 26.8		
Age (years)			0.377	
0–5	11 (20.4%)	24 (22.2)		
6–11	2 (3.7%)	0 (0.0%)		
12–18	2 (3.7%)	8 (7.4%)		
19–26	1 (1.9%)	4 (3.7%)		
27–59	21 (38.9%)	40 (37.0%)		
60 and more	17 (31.5%)	32 (29.6%)		
Type of HAIs			0.161	
VAP	27 (50.0%)	54 (50.0%)		
CLABSI	16 (29.6%)	32 (29.6%)		
SSI	5 (9.3%)	10 (9.3%)		
CAUTI	5 (9.3%)	10 (9.3%)		
NV-HAP	1 (1.9%)	2 (1.9%)		
Comorbidities			0.102	
Hypertension	24 (44.4%)	36 (33.3%)	0.173	1.600
Diabetes mellitus	13 (24.1%)	26 (24.1%)	1.000	1.000
Oncohaematological diseases	9 (16.7%)	12 (11.1%)	0.331	1.600
Underweight	9 (16.7%)	9 (8.3%)	0.120	2.200
Cardiopathy	3 (5.6%)	11 (10.2%)	0.389	0.519
Chronic kidney disease	3 (5.6%)	5 (4.6%)	1.000	1.212
Rheumatological diseases	2 (3.7%)	1 (0.9%)	0.258	4.115
Obesity	2 (3.7%)	1 (0.9%)	0.258	4.115
Cancer	1 (1.9%)	5 (4.6%)	0.665	0.389
Asthma	1 (1.9%)	2 (1.9%)	1.000	1.000
HIV	1 (1.9%)	1 (0.9%)	1.000	2.019
Invasive Procedures				
Central venous catheter	53 (98.1%)	97 (89.8%)	0.063	6.01
Urinary catheter	43 (79.6%)	75 (69.4%)	0.193	1.72
Mechanical ventilation	41 (75.9%)	63 (58.3%)	0.37	2.253
Avg. invasive procedures (days)				
Central venous catheter	14.2	10.2	0.005	
Urinary catheter	9.3	6.3	0.101	
Mechanical ventilation	9.0	6.6	0.150	
Avg. of hospital stays (days)	40.2	36.9	0.122	
Avg. of ICU stays (days)	21.4	22.4	0.196	
Outcome			0.360	
Hospital discharge	31 (57.4%)	65 (60.2%)		
Death	20 (37.0%)	31 (28.7%)		
Reference to 2nd. level hospital	3 (5.6%)	12 (11.1%)		
Bacteria				
*Acinetobacter baumannii*	54 (100%)	--		
*Escherichia coli*	--	30 (27.8%)		
*Pseudomonas aeruginosa*	--	28 (25.9%)		
*Klebsiella pneumoniae*	--	27 (25.0%)		
*Staphylococcus aureus*	--	16 (14.8%)		
*Enterococcus faecalis*	--	7 (6.5%)		
Drug-resistant Profile				
*Acinetobacter baumannii*				
MDR	5 (9.3%)	--		
XDR	49 (90.7%)	--		
*Escherichia coli*				
MDR	--	5 (4.65%)		
XDR	--	26 (24.1%)		
*Pseudomonas aeruginosa*				
MDR	--	17 (15.8%)		
XDR	--	11 (10.1%)		
*Klebsiella pneumoniae*				
MDR	--	4 (3.7%)		
XDR	--	22 (20.3%)		
*Staphylococcus aureus*				
MDR	--	5 (4.6%)		
XDR	--	11 (10.3%)		
*Enterococcus faecalis*				
MDR	--	4 (3.7%)		
XDR	--	3 (2.8%)		
Previous Antibiotic Therapy				
Penicillins: AMP	6 (11.1%)	7 (6.5%)		
1st gen. CP: CET	0 (0.0%)	3 (2.8%)		
2nd gen. CP: CRX	0 (0.0%)	6 (5.6%)		
3rd gen. CP: CTX, CRO, CAZ	18 (33.3%)	45 (41.7%)	0.143	
4th gen. CP: FEP	6 (11.1%)	9 (8.3%)	0.565	
Carbapenems: MEM, IMP	12 (22.2%)	28 (25.9%)	<0.000	
Monobactams: PIP-TZ	7 (13.0%)	7 (6.5%)	0.166	
Aminoglycosides: AMK, GEN	15 (27.8%)	20 (18.5%)	0.177	
Glycopeptides: VAN	14 (25.9%)	18 (16.7%)	0.163	
Macrolides: CLI	4 (7.4%)	5 (4.6%)	0.467	
FQs: LEV, CIP, MXF	29 (53.7%)	42 (32.4%)	0.009	
Oxazolidinones: LZD	13 (24.1%)	5 (4.6%)	<0.000	
No. of Antibiotics Prescribed			0.002	
Cero	0	9 (9.2%)		
One	12 (22.2%)	47 (47.5%)		
Two	15 (27.8%)	28 (28.3%)		
Three	17 (31.5%)	15 (15.2%)		
Four	5 (9.3%)	8 (8.1%)		
Five to Seven	5 (9.3%)	1 (1.0%)		

Statistical significance *p* < 0.05. VAP: Ventilator-associated pneumonia; CLABSI: Central line-associated bloodstream infections; SSI: Surgical site infections; CAUTI: Catheter-associated urinary tract infections; NV-HAP: Non-ventilator hospital-acquired pneumonia; HIV: Human immunodeficiency virus; FQs: Fluoroquinolones; LEV: Levofloxacin; CIP: Ciprofloxacin; MXF: Moxifloxacin; MEM: Meropenem; IMP: Imipenem; CP: Cephalosporin; CTX: Cefotaxime; CRO: Ceftriaxone; CAZ: Ceftazidime; AMK: Amikacin; GEN: Gentamicin; VAN: Vancomycin; LZD: Linezolid; PIP-TZ: Piperacillin and Tazobactam; FEP: Cefepime; AMP: Ampicillin; CLI: Clindamycin; CRX: Cefuroxime; CET: Cephalothin.

## Data Availability

The original contributions of this study are comprehensively detailed within the article. For further inquiries, please contact the corresponding author.
